# Anti-arthritic and toxicological evaluation of ethanolic extract of *Alternanthera bettzickiana* in rats

**DOI:** 10.3389/fphar.2022.1002037

**Published:** 2022-10-24

**Authors:** Maria Manan, Uzma Saleem, Bashir Ahmad, Nosheen Aslam, Asifa Anwar, Aimen Zafar

**Affiliations:** ^1^ Department of Pharmacology, Faculty of Pharmaceutical Sciences, Government College University Faisalabad, Faisalabad, Pakistan; ^2^ Hamza College of Pharmaceutical and Allied Health Scieces, Lahore, Pakistan; ^3^ Deparment of Biochemistry, Government College University Faisalabad, Faisalabad, Pakistan; ^4^ Department of Pharmacy, Lahore Medical and Dental College, Lahore, Pakistan; ^5^ University Institute of Food Science and Technology, University of Lahore, Lahore, Pakistan

**Keywords:** rheumatoid arthritis, *Alternanthera bettzickiana*, acute oral toxicity, ELISA, C-reactive protein

## Abstract

In many developing countries, medicinal plants have long been used for therapeutic purposes due to their low cost and toxicity. This study evaluated the safety and anti-arthritic potential of *Alternanthera bettzickiana* ethanolic extract (ABEE)*.* Acute oral toxicity (OECD 425) was tested in the safety evaluation. A limit test was used to identify the LD_50_ value. For an acute oral toxicity study a dose of 2000 mg/kg of ABEE was given orally to the treatment group, and the control group received distilled water at a rate of 10 ml/kg. Biochemical, hematological, and histopathological analyses were performed after 14 days. A formaldehyde 2% w/v solution was injected *via* i.p. to rats of all groups to prepare the arthritic model. Five groups were divided into control (D.H_2_O), standard (Diclofenac), and three groups receiving the plant extract at dose levels of 125 mg/kg, 250 mg/kg, and 500 mg/kg respectively. Treatment was continued for 10 days. Paw diameter and hematological and biochemical variables were quantified. ELISA was performed for the estimation of inflammatory cytokines. In the acute oral toxicity study, no mortality or morbidity were observed, so the LD_50_ of this plant was greater than 2000 mg/kg. ABEE decreased the paw diameter with the restoration of hematological and biochemical changes. SOD and CAT levels were increased while decreasing the MDA, NO, TNF-α, and IL-6 levels in arthritic rats. It is concluded that the use of *A. bettzickiana* has low toxicity, and it can be used for the treatment of arthritis.

## Graphical Abstract

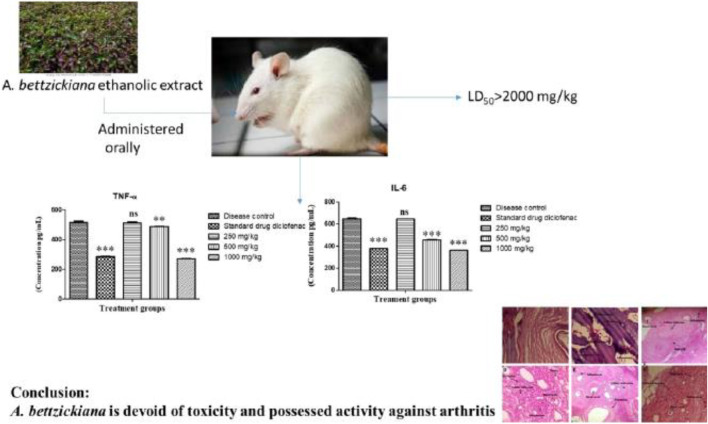

## 1 Introduction

The traditional use of herbal medicines was common before the evolution of synthetic and semisynthetic medicines and remains common (Giaid et al., 1993; Rahman and Gulshana, 2014; Jain et al., 2021). Many populations in developing countries depend upon herbal remedies because they are accessible and highly trusted therapy. Their use is increasing due to their availability, efficacy, and patient acceptability (Patil and Gaikwad, 2010; Benzie and Wachtel-Galor, 2011). These therapies possess natural potentials that help in competing disorders, such as arthritis, renal and hepatic disorders, and obesity (Kunle et al., 2012). Herbal preparations are taken by approximately 12% of the population in the United States in 1993 (Eisenberg et al., 1993; Eisenberg et al., 1998). It is generally thought that herbal therapies do not show side effects, in contrast to synthetic medications (Latha et al., 2010; Li and Wang, 2021).

The efficacies of herbal plants are rarely tested, despite of their significant use (Calixto, 2000; Unuofin et al., 2018). The safety of herbal remedies has become a prime focus of researchers examining herbal remedies (Chen et al., 2006). It is important to standardize the herbal preparations utilized in the treatment of various disorders (Hunter, 2008; Unuofin et al., 2018). Plants possessing biological activity should show less toxicity due to long-term use. However, many traditional remedies used in traditional medicines have shown toxic effects (Ertekin et al., 2005; Koduru et al., 2006). Paracelsus, the father of toxicology, said that all substances are poisons, but it is the dose that differentiates between treatment and poison (Hunter, 2008). Many medicines are produced from herbal remedies. Many of these preparations depend upon the use of agents in folkloric medicines. Rheumatoid arthritis is a systemic inflammatory autoimmune disease with no cure. It is marked by the consistent swelling of the synovial joints, which gives rise to bone and cartilage erosion. The objective of therapy is to alleviate joint damage, conserve function, and avert disability. RA can cause irreversible joint destruction in untreated patients within 2 years (Lee et al., 2021). Moreover, initial joint destruction in patients with recent onset could be associated with later disability (Maillefert et al., 2004). Ultimately, several organs may be affected. The main targets of disease are joints but it can affect other systems of body. *Alternanthera bettzickiana* belongs to the Amaranthaceae family. Amaranthaceae includes nearly 65 genera and more than 900 species. The family is widely distributed across tropical regions. Plants in this family are used for ornamental purposes, like *A. salixifolius*, *Celosia cristata*, *Iresine herbtsii*, etc., and some are edible and used as vegetables, such as *A. hybridus*, *A. spinosus*, *A. tricolor*, *A. viridis*, etc. (Rahman and Gulshana, 2014). *A. bettzickiana* has been cultivated in many places across the world and is native to South America. It is also called the border plant, the red calico, and the Baptist plant. Locally in Pakistan, it is also called *nanthra*. It is frequently utilized as a bordering plant, with a food and ornamental use. Its leaves can be green, red, or both. Its leaves are used as vegetable and spinach. Its leaves are also cooked with vegetables and served with rice (Pamila and Karpagam, 2017a). It possesses wound healing, soft laxative, blood purifying, galactagogue, and fever-reducing properties. Traditionally, it is used for reducing gastrointestinal discomfort and the prophylaxis of dementia and in the treatment of arthritis in Thailand (Álvarez, 2009; Maneenoon et al., 2015). It also possess anti-Alzheimer’s, antimicrobial, diuretic, hemolytic, cytotoxic, anti-inflammatory, and mutagenic activities (Barksby et al., 2007; Phusricom et al., 2013; Vidhya et al., 2015; Akhtar et al., 2017; Pamila and Karpagam, 2017a). This study was carried out to assess the acute toxicity and anti-arthritic activity of *A. bettzickiana* using a formaldehyde-induced arthritis model.

## 2 Materials and methods

### 2.1 Collection

The aerial parts of the plant were collected from Punjab in March 2019. Plant identification and authentication was performed by Dr. Mansoor, Department of Botany, University of Agriculture Faisalabad (UAF), and voucher no. 520-1-13 was issued for a crude sample of UAF herbarium.

### 2.2 Preparation of extract

Microwave extraction technology was used for the preparation of the extract. The extraction procedure involves three cycles after setting the microwave to 9,000 W. In the first cycle, 750 ml ethanol is used, while in the other two cycles, 500 ml ethanol was added to 100 g powder in three beakers. Beakers were placed into the microwave oven and cooked for 2 min. The microwave oven door remained open for a period of 30 s. This procedure was repeated five times. The same process was repeated for other cycles. The extract was filtered by muslin cloth and then by whatman filter paper to obtain filtrate. The filtrate from every cycle was collected, and the solvent was evaporated at 40°C. The extract was kept in amber colored bottles (Shah et al., 2017).

### 2.3 Animal husbandry

Following the OECD guideline 425, nonpregnant female Wistar rats weighing 130 ± 30 g and aged 9–10 weeks were randomly taken. Rats were housed under standard conditions for 5 days to acclimatize them. The animal house was kept at 22 ± 3°C with 30–70% relative humidity. The light and dark cycle was 12 h long. The rats were given a standard laboratory diet with tap water.

### 2.4 Approval from animal’s ethics committee

The study was carried out after taking permission from animal’s ethics committee, Govt. College University Faisalabad with Ref. No. GCUF/ERC/2143.

### 2.5 Acute oral toxicity study (OECD 425)

The rats were housed under standard conditions in the animal house of Govt. College University Faisalabad (GCUF) for 5 days to acclimatize them with new environment. The limit test dose of 2000 mg/kg dose was given. Rats were fasted 3–4 h before dosing but provided with water. Dosing was given to single rats on the basis of weight. Rats were strictly monitored for initial 30 min and then for 4 hours. Food was given 1–2 h after dosing. Four rats were given the same dose orally after survival of first rat. The same procedure was adopted for control group of five rats. The control group was administered distilled water. Both groups were observed for the toxic effects for first 6 h and then for a duration of 14 days. The surviving rats were noticed for any toxic reaction. The weight of animals was recorded as well. After 14 days rats were again weighed and blood was accumulated *via* cardiac puncture under chloroform anesthesia. The serum was isolated for carrying out biochemical and hematological analysis. Vital organs (liver, heart, and kidney) were removed from slaughtered rats after cervical dislocation. The weight of these organs was recorded. The organs were preserved in 10% formaldehyde solution and were implanted in paraffin wax (Saleem et al., 2017).

#### 2.5.1 Hematological and biochemical analysis

Blood from both treated and vehicle control rats was collected in EDTA-containing tubes for hematological analysis. Hemoglobin (Hb), total RBC, white blood cells (WBC) count, platelets, mean corpuscular volume (MCV), packed cell volume (PVC), monocytes (M), neutrophils (N), mean corpuscular hemoglobin (MCH), eosinophils (E), mean corpuscular hemoglobin concentration (MCHC), and lymphocytes (L) were determined using Mythic CBC analyzer. Serum was separated for biochemical analysis. Cholesterol, triglycerides, low density lipoprotein (LDL), high density lipoprotein (HDL), very low density lipoprotein (VLDL), bilirubin, alanine aminotransferase (ALT), aspartate aminotransferase (AST), alkaline phosphatase, urea, blood urea nitrogen, creatinine, uric acid, proteins, albumin, globulin, A/G ratio, rheumatoid factor (RF), and C-reactive protein (CRP) were measured using Tecno 786 bio chemistry analyzer (Anwar et al., 2021b).

#### 2.5.2 Histopathological analysis

Vital organs separated from slaughtered rats were fixed in formalin (10%) and then implanted in paraffin wax. Sections were slashed at 5 mm and stained with hematoxylin and eosin. Histopathological modifications were studied under a photomicroscope (Anwar et al., 2021a).

### 2.6 *In vivo* assessment of anti-arthritic potential by formaldehyde-induced arthritis

Wistar albino rats weighing 150–180 g were used. Rats were divided into five groups of five rats each. Group 1 (disease control group): rats were injected with formaldehyde solution on the first and third day of experiment. Group 2 (Diclofenac sodium treated): rats were injected with formaldehyde solution, as in group 1, and administered diclofenac sodium daily at a dose of 10 mg/kg for 10 days. Groups 3–5 (ABEE treated) rats received formaldehyde solution as in group 1 and were administered ABEE at a dose of 250, 500, 1,000 mg/kg, respectively, for 10 days. All treatments were dissolved in distilled water and given orally; 30 min after drug administration, arthritis was developed by a sub-plantar injection of 0.1 ml of 2% w/v formaldehyde solution on days 1 and 3 of the experiment (Uttra et al., 2019). Arthritis was assessed by measuring mean increase in paw diameter by Vernier caliper for 10 days. The percentage inhibition of edema was calculated utilizing the following formula:

$$\%\;inhibition=\frac{Vc-Vt}{Vt}*100$$ (1)

where Vc is the paw diameter of disease control and Vt is the paw diameter of treated.

#### 2.6.1 Arthritis evaluation by hematological and biochemical parameters

The rats were slaughtered under chloroform anesthesia after 10 days of study, and blood was collected *via* cardiac puncture. The hematological factors (red blood cells, hemoglobin, white blood cells, erythrocyte sedimentation rate, and platelet count) were measured by Mythic CBC analyzer while biochemical variables (alanine aminotransferase, alkaline phosphatase, aspartate aminotransferase, creatinine, rheumatoid factor, C-reactive protein, and urea) were determined with Tecno 786 bio chemistry analyzer. Next, animals were sacrificed by painless procedure by cervical dislocation under anesthesia (Saleem et al., 2020a).

#### 2.6.2 Enzyme linked immunosorbent assay

ELISA was performed to determine the concentrations of tumor necrosis factor and interleukin 6 in serum using kit protocols (Elabscience, Catalog Number E-EL-R0019 and E-EL-R0015) (Cheng et al., 2015).

#### 2.6.3 Determination of oxidative stress biomarkers

The serum concentration of nitric oxide was measured using Nitric Oxide ELISA kit (Elabscience, Catalog number E-BC-K036). Microplate ELISA Reader (BI, United States, 800TS-UV) was used to measure the absorbance. Serum levels of superoxide dismutase (Elabscience, Catalog number E-BC-K020), catalase (Elabscience, Catalog number E-BC-K106), and malondialdehyde (Elabscience, Catalog number E-EL-0060) were determined after the study ended (Saleem et al., 2020b).

### 2.7 Statistical analysis

The results were presented as means ± SEMs (*n* = 5), and the statistical significance was analyzed by two-way ANOVA, followed by Bonferroni’s posttest, and applied by Graph Pad Prism 5 software. *p* ≤ 0.05 was considered to indicate statistical significance.

## 3 Results

### 3.1 Acute oral toxicity study

#### 3.1.1 Body weight and behavioral changes

The administration of ABEE showed no mortality throughout the study. Experimental animals were observed for 14 days with special consideration. The body weights of vehicle control and ABEE-treated rats were increased, as shown in Table 1. Slight behavioral changes were recorded in treated rats, such as convulsions, and tremor was recorded in ABEE treated rats after during initial 3 h. Any effect on the fur of the treated animals were elevated for 2 h after dosing. Somatomotor activity was decreased during experimental period. The respiratory rate was also increased in extract treated rats for one and half hour after dosing then it became normal. Itching was observed during first week of study in both groups. Other behavioral features were normal compared with vehicle control rats (Table 2).

**TABLE 1:** Effect of ABEE (at limit dose 2000 mg/kg p.o.) and vehicle treatment on body weight of rats in acute toxicity study.

Groups	Body weight on 1st day (g)	Body weight on 14th day (g)
Control group (Distilled water)	141.94 ± 2.24	145 ± 3.80^ns^
Treatment group (ABEE 2000 mg/kg)	132 ± 6.27	138 ± 2.91^ns^

Values are presented as means ± SEMs (*n* = 5) and analyzed with two-way ANOVA followed by a Bonferroni posttest. ns, nonsignificant as compared to disease control. ABEE, *Alternanthera bettzickiana* ethanolic extract.

**TABLE 2:** Behavioral pattern of rats treated with ABEE (2000 mg/kg p.o.) during acute toxicity study.

Parameters	Observations of vehicle control and ABEE-treated group											
	30 min		4 h		24 h		48 h		7 days		14 days	
	VCG	ABTG	VCG	ABTG	VCG	ABTG	VCG	ABTG	VCG	ABTG	VCG	ABTG
Eyes	N	N	N	N	N	N	N	N	N	N	N	N
Respiration	N	↑	N	N	N	N	N	N	N	N	N	N
Itching	P	P	P	P	P	P	P	P	P	P	NF	NF
Sleep	N	N	N	N	N	N	N	N	N	N	N	N
Fur	N	E	N	N	N	N	N	N	N	N	N	N
Urination	N	N	N	N	N	N	N	N	N	N	N	N
Gait	N	SI	N	SI	N	SI	N	SI	N	N	N	N
Convulsion/tremor	NF	P	NF	P	NF	P	NF	P	NF	NF	NF	NF
Somatomotor activity	N	MD	N	MD	N	MD	N	MD	N	MD	N	N
Salivation	N	N	N	N	N	N	N	N	N	N	N	N
Coma	NF	NF	NF	NF	NF	NF	NF	NF	NF	NF	NF	NF
Feces consistency	N	N	N	N	N	N	N	N	N	N	N	N
Mucous membrane	N	N	N	N	N	N	N	N	N	N	N	N
Mortality	NF	NF	NF	NF	NF	NF	NF	NF	NF	NF	NF	NF

CG, control group; ABTG, *Alternanthera bettzickiana* treated group; N, normal; E, elevated; P, present; SI, slightly elevated; ↑, increased; MD, moderately decreased; NF, not found.

#### 3.1.2 Organ-to-body weight index

Organ-to-body weight index was determined and is presented in Table 3; it showed no remarkable changes among the groups. No lesions were observed on isolated organs, such as the liver, heart, or kidney, of the treated animals.

**TABLE 3:** Effect of ABEE (at limit dose 2000 mg/kg p.o.) and vehicle treatment on weight of organs and organ-to-body weight indices in acute toxicity study.

Groups	Heart	Kidney	Liver
Weight of organs (g)			
Control group (distilled water)	0.67 ± 0.01	0.51 ± 0.03	4.16 ± 0.02
Treatment group (ABEE 2000 mg/kg)	0.58 ± 0.01^ns^	0.49 ± 0.01^ns^	4.20 ± 0.13^ns^
Organ-to-body weight indices			
Control group (distilled water)	0.462 ± 0.027	0.351 ± 0.017	2.868 ± 0.029
Treatment group (ABEE 2000 mg/kg)	0.420 ± 0.025 ^ns^	0.355 ± 0.010 ^ns^	3.043 ± 0.010 ^ns^

Values are presented as means ± SEMs (*n* = 5) and analyzed by two-way ANOVA followed by a Bonferroni posttest. ns, nonsignificant as compared to disease control. ABEE, *Alternanthera bettzickiana* ethanolic extract; organ-to-body weight index = (organ weight ×100)/body weight.

#### 3.1.3 Effect of ABEE on hematological and biochemical variables in acute oral toxicity study

The hematological and biochemical profiles of rats treated with ABEE are shown in Tables 4 and 5. No significant change was observed for hemoglobin, RBCS, ESR and TLC, neutrophils, eosinophils, lymphocytes, or monocytes, in contrast to vehicle control group. It should also be noted that there were no significant alterations in HCT, MCHC, MCH, or MCV in comparison to vehicle control rats. The platelet count was raised significantly in the treatment group (*p* < 0.001) when compared with the vehicle control group. It was noted that rats showed no significant changes in LDL, VLDL, cholesterol, HDL, or triglycerides values of the treatment group relative to the vehicle control group values (Table 5). The levels of aspartate aminotransferase (AST), alanine aminotransferase (ALT), proteins, globulin, albumin, A/G ratio were altered nonsignificantly in the treatment group in contrast to vehicle control rats. Nonsignificant variation was recorded in the urea, blood urea nitrogen, creatinine, uric acid, and total bilirubin levels of treatment rats when compared with vehicle control rats (Table 5). Alkaline phosphatase and protein levels were decreased significantly in the treatment group relative to the vehicle control group.

**TABLE 4:** Effect of ABEE (at limit dose 2000 mg/kg p.o.) on hematologic variables in acute toxicity study.

Parameters	Units	Control group	Treatment group (2000 mg/kg)
Hemoglobin	g/dL	15.2 ± 1.14	16.8 ± 0.86^ns^
TLC	*10^9^/L	6.3 ± 0.76	7.3 ± 0.57^ns^
ESR	mm/1st h	6 ± 1.1.4	7.4 ± 0.71^ns^
RBC	*10^12^/L	6.57 ± 0.67	7.14 ± 0.93^ns^
HCT (PCV)	%	37.2 ± 1.33	36.6 ± 0.94^ns^
MCV	Fl	62.8 ± 0.64	58.3 ± 2.91^ns^
MCH	Pg	25.4 ± 2.70	26.4 ± 0.49^ns^
MCHC	%	53.2 ± 2.28	48.2 ± 0.60^ns^
Platelets	*10^9^/L	937 ± 11.7	1,437 ± 8.95***
Neutrophils	%	4 ± 0.35	5 ± 0.35^ns^
Lymphocytes	%	90 ± 9.06	93 ± 5.59^ns^
Monocytes	%	5 ± 0.35	2 ± 0.48^ns^
Eosinophils	%	1 ± 0.22	2 ± 0.48^ns^
RF	IU/mL	23.6 ± 1.34	19.62 ± 0.97^ns^
CRP	mg/L	3.70 ± 0.65	2.80 ± 0.58^ns^

Values are presented as means ± SEMs (*n* = 5) and analyzed by two-way ANOVA followed by a Bonferroni posttest. ns, nonsignificant, ***p < 0.001 as compared to disease control. ABEE, *Alternanthera bettzickiana* ethanolic extract.

**TABLE 5:** Effect of ABEE (at limit dose 2000 mg/kg p.o.) on biochemical variables in an acute toxicity study.

Parameters	Units	Control group	Treatment group (2000 mg/kg)
Lipid profile			
Cholesterol	mg/dL	81 ± 1.70	74 ± 3.25^ns^
Triglycerides	mg/dL	97 ± 4.49	86 ± 5.40^ns^
High density lipoprotein	mg/dL	24 ± 4.52	23 ± 2.12^ns^
Low density lipoprotein	mg/dL	73 ± 6.21	66 ± 4.95^ns^
Very low density lipoprotein	mg/dL	19 ± 2.28	17 ± 2.02^ns^
Liver function test			
Bilirubin	mg/dL	0.37 ± 0.01	0.34 ± 0.01^ns^
AST	U/L	63 ± 6.51	57 ± 6.51^ns^
ALT	U/L	52 ± 6.96	48 ± 3.42^ns^
Alkaline phosphatase	U/L	216 ± 8.34	143 ± 6.58***
Albumin	g/L	3.30 ± 0.02	2.90 ± 0.03^ns^
Globulin	g/L	2.9 ± 0.02	2.4 ± 0.02^ns^
Proteins	g/L	6.20 ± 0.53	5.30 ± 0.60***
A/G ratio	g/L	1.1 ± 0.007	1.20 ± 0.01^ns^
Renal function test			
Urea	mg/dL	18 ± 2.02	16 ± 2.02^ns^
BUN	mg/dL	10 ± 1.14	9 ± 0.70^ns^
Creatinine	mg/dL	0.56 ± 0.02	0.41 ± 0.01^ns^
Uric acid	mg/dL	3.20 ± 0.44	2.90 ± 0.03^ns^

Values are presented as means ± SEMs (*n* = 5) and analyzed by two-way ANOVA followed by a Bonferroni posttest. ns, nonsignificant, ***p < 0.001, as compared to disease control. ABEE, *Alternanthera bettzickiana* ethanolic extract.

#### 3.1.4 Histopathological assessment in acute oral toxicity study

**FIGURE 1:**
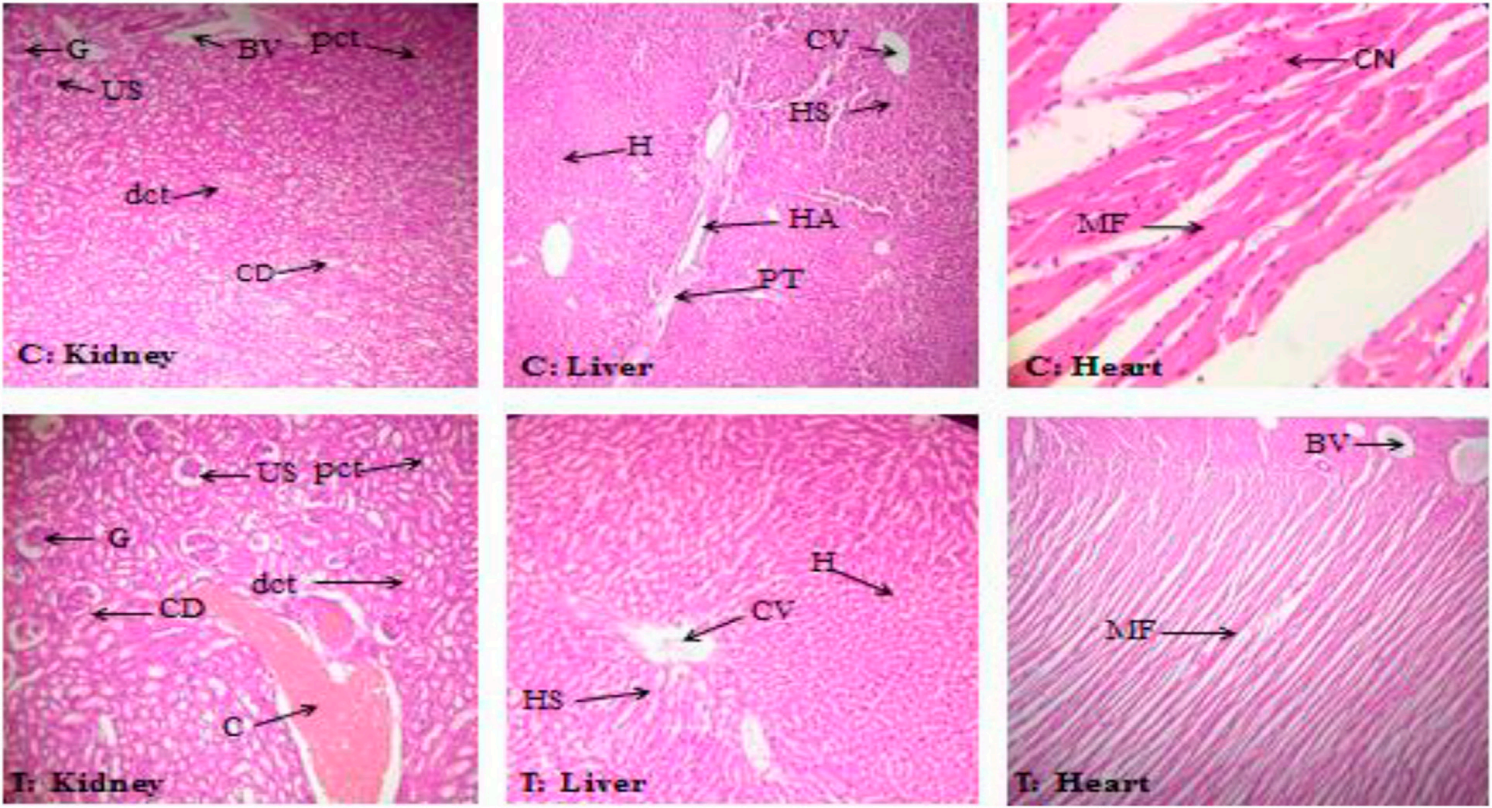
Histopathological assessment of kidney, liver, and heart of the control group (C) and the treatment group (T) at 40 × and 10 × magnification. G: glomerulus; US: urinary space; BV: blood vessel; pct: proximal convoluted tubules; dct: distal convoluted tubules; CD: collecting duct; C: congestion; H: hepatocytes; CV: central vein; HS: hepatic sinusoid; HA: hepatic artery; PT: portal triad; CN: central nuclei; MF: myocardial fibrils.

Histopathological assessment showed no structural changes in the kidney, liver, or heart tissue of ABEE-treated rats. Histopathological assessment showed that ABEE did not lead to any side effects on physiological features, and the architecture of the vehicle control and acute toxicity groups was found to be similar, with only slight congestion being recorded in the kidney tissues of treatment rats (Figure 1). The overall histopathological assessment of kidney liver and heart sections of ABEE-treated rats showed no remarkable variation in contrast to vehicle control group, which means that the plant was safe at a 2000 mg/kg dose.

### 3.2 Effect of *Alternanthera bettzickiana* ethanolic extract in formaldehyde-induced arthritis in Wistar rats

#### 3.2.1 Effect on paw diameter

Table 6 sums up the effects of ABEE (250, 500, 1,000 mg/kg) and diclofenac sodium (10 mg/kg) in formaldehyde-induced arthritis. Formaldehyde injection showed a swelling of left hind paw of all rats on the first and third days. The increase in paw inflammation was recorded for 4 days, and then it was found attenuating in treated rats in a concentration dependent manner. ABEE at a 1,000 mg/kg dose exhibited maximum inhibitory effect (72.11%) on paw diameter, while 500 and 250 mg/kg extract revealed a 65.25% and 56.62% reduction in paw diameter respectively. Diclofenac sodium (10 mg/kg) rats showed 70.80% decrease (*p* < 0.001) in paw diameter at day 10. These results indicated that ABEE at 1,000 mg/k exhibited a peak effect, and the outcomes were prominent relative to the standard drug diclofenac sodium.

**TABLE 6:** Effects of ABEE on paw diameter of formaldehyde-induced arthritic rats.

Treatment groups	Paw diameter					
	Day 0	Day 2	Day 4	Day 6	Day 8	Day 10
Disease control	3.83 ± 0.32	5.84 ± 0.55	7.86 ± 0.539	9.97 ± 0.316	10.51 ± 0.628	12.98 ± 0.412
Standard drug diclofenac (10 mg/kg)	3.44 ± 0.184^ns^	4.95 ± 0.270^ns^ (15.23%)	5.91 ± 0.283** (24.80%)	5.42 ± 0.212*** (45.63%)	4.34 ± 0.298*** (58.70%)	3.79 ± 0.201*** (70.80%)
ABEE 250 m/kg	3.74 ± 0.292^ns^	5.54 ± 0.595^ns^ (5.13%)	6.83 ± 0.430^ns^ (13.10%)	6.41 ± 0.607*** (35.70%)	5.72 ± 0.354*** (45.57%)	5.63 ± 0.336*** (56.62%)
ABEE 500 mg/kg	3.69 ± 0.116^ns^	5.19 ± 0.20^ns^ (11.13%)	6.26 ± 0.519* (20.35%)	5.93 ± 0.604*** (40.52%)	4.94 ± 0.185*** (52.99%)	4.51 ± 0.212*** (65.25%)
ABEE 1000 mg/kg	3.69 ± 0.311^ns^	4.58 ± 0.58^ns^ (21.57%)	5.47 ± 0.342*** (30.40%)	4.64 ± 0.381*** (53.46%)	3.85 ± 0.430*** (63.36%)	3.62 ± 0.130*** (72.11%)

Results are presented as means ± SEMs (*n* = 5) and analyzed by two-way ANOVA followed by a Bonferroni posttest. ns, nonsignificant, ***p < 0.001, as compared to disease control. ABEE, *Alternanthera bettzickiana* ethanolic extract.

#### 3.2.2 Effect on hematological and biochemical variables

The blood and serum parameters of animals administered with ABEE and diclofenac sodium are shown in Table 7. Changes in these parameters were evaluated after 10 days of formaldehyde induction in rats of various groups. Hematological and biochemical variables, such as elevation in ALP, AST, ALT, platelets, WBCs count, creatinine, CRP, urea, and RF, in addition to reduction in RBCs and hemoglobin, were observed in disease control group. Similarly, administration with ABEE and standard drug diclofenac sodium significantly (*p* < 0.001) averted abnormal modifications of hematological variables in treatment groups altered by the formaldehyde injection. Furthermore, ALP, ALT, and AST levels noticeably increased in disease control rats and significantly decreased in animals treated with 500 and 1,000 mg/kg ABEE, while 250 mg/kg dose of extract exhibited nonsignificant decrease in ALP, ALT, and AST levels in treatment group relative to the disease control group. Similarly, the standard drug diclofenac sodium also significantly decreased ALP, ALT, and AST levels in treated group, by contrast to disease control group. Moreover, the noticeably increased creatinine and urea concentrations in disease control animals were significantly decreased with ABEE and standard drugs in treatment rats. Additionally increased serum values of CRP and RF were observed in disease control animals, while administration with ABEE and standard drug diclofenac sodium significantly decreased CRP and RF levels in treatment groups. These results indicated that ABEE at 1,000 mg/k exhibited peak effects, and outcomes were prominent as compared to standard drug diclofenac sodium.

**TABLE 7:** Effects Of ABEE on hematological and biochemical variables of formaldehyde-induced arthritic rats.

Parameters	Disease control	Standard (10 mg/kg)	ABEE (250 mg/kg)	ABEE (500 m/kg)	ABEE (1,000 mg/kg)
Hb	4.59 ± 0.43	15.23 ± 1.03***	10.51 ± 1.84*	12.31 ± 1.41**	16.10 ± 1.07***
ESR	17 ± 1.07	8.00 ± 1.41**	14 ± 2.12^ns^	11 ± 1.41*	7 ± 1.17***
RBCs	4.58 ± 0.48	10.91 ± 0.55***	5.99 ± 0.58^ns^	9.88 ± 1.14**	11.78 ± 1.30***
WBCs	19.63 ± 1.68	10.38 ± 1.04***	18.6 ± 0.88^ns^	13.59 ± 1.41**	7.57 ± 0.34***
Platelets	1958 ± 19.14	972.80 ± 8.06***	1,343 ± 17.82***	1,153 ± 11.60***	851 ± 5.00***
AST	78.75 ± 3.68	47.90 ± 3.70***	75.90 ± 4.56^ns^	59.78 ± 0.44**	31.60 ± 2.30***
ALP	254 ± 8.78	105.23 ± 3.80***	253.65 ± 6.51^ns^	200.56 ± 17.40**	98.38 ± 4.11***
ALT	48 ± 5.59	25 ± 1.58***	37 ± 1.14^ns^	30 ± 1.61**	17 ± 1.84***
Urea	42.80 ± 1.51	17 ± 1.41***	32 ± 1.41***	21 ± 0.74***	15 ± 1.84***
Creatinine	4.3 ± 0.277	2.40 ± 0.44**	3.60 ± 0.114^ns^	2.50 ± 0.412**	1.8 ± 0.326***
CRP	21.3 ± 2.70	12.78 ± 1.30*	16.51 ± 1.58^ns^	10.53 ± 1.84**	7.54 ± 1.84***
RF	22 ± 1.19	10 ± 1.70***	15.04 ± 1.01**	13 ± 0.65***	9 ± 2.00***

Results are presented as means ± SEMs (*n* = 5) and analyzed by one-way ANOVA followed by a Bonferroni posttest. ns, nonsignificant; ***p < 0.001; **p < 0.01; *p < 0.05 as compared to disease control. Hb, hemoglobin; ESR, erythrocyte sedimentation rate; RBCs, red blood cells; WBCs, white blood cells; AST, aspartate aminotransferase; ALP, alkaline phosphatase; ALT, alanine aminotransferase; CRP, C-reactive protein; RF, rheumatoid factor; BUN, blood urea nitrogen; HDL, high-density lipoprotein; LDL, low-density lipoprotein; ABEE, *Alternanthera bettzickiana* ethanolic extract.

#### 3.2.3 Effect on serum levels of TNF-α and IL-6: ELISA study

**FIGURE 2:**
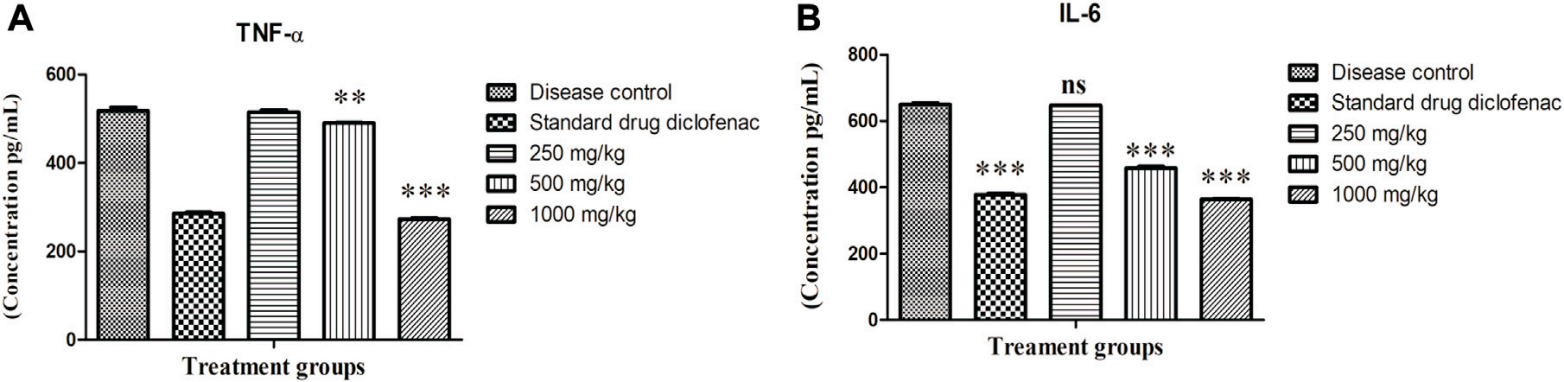
Effect of *Alternanthera bettzickiana* ethanolic extract on inflammatory mediators **(A)** TNF-α and **(B)** IL-6 in formaldehyde-induced arthritis. Results are presented as means ± SEMs (*n* = 5) and analyzed by one-way ANOVA followed by a Bonferroni posttest. ns, nonsignificant; ***p < 0.001; **p < 0.01 as compared to disease control. ABEE, *Alternanthera bettzickiana* ethanolic extract.

An elevated level of TNF-α (*p* < 0.001) was noticed in disease control rats (517.59 ± 8.355 pg/ml), although the elevation was significantly decreased by administration of ABEE at 1,000 mg/kg (273.45 ± 2.30 pg/ml), 500 mg/kg (490.00 ± 1.70 pg/ml) and diclofenac sodium (285.79 ± 2.91 pg/ml) when compared with disease control rats (Figure 2A). An abnormally increased level of IL-6 was noted in the serum of the disease control group (649.91 ± 4.52 pg/ml). This elevated level was significantly (*p* < 0.001) decreased by ABEE at 1,000 mg/kg (363.50 ± 2.31 pg/ml), 500 mg/kg (457.53 ± 5.70 pg/ml), and diclofenac sodium (377.91 ± 2.98 pg/ml) (Figure 2B). The most effective dose of plant extract, which, exhibited the highest decrease in serum concentration of TNF-α and IL-6, was 1,000 mg/kg.

#### 3.2.4 Effect on oxidative stress biomarkers

**FIGURE 3:**
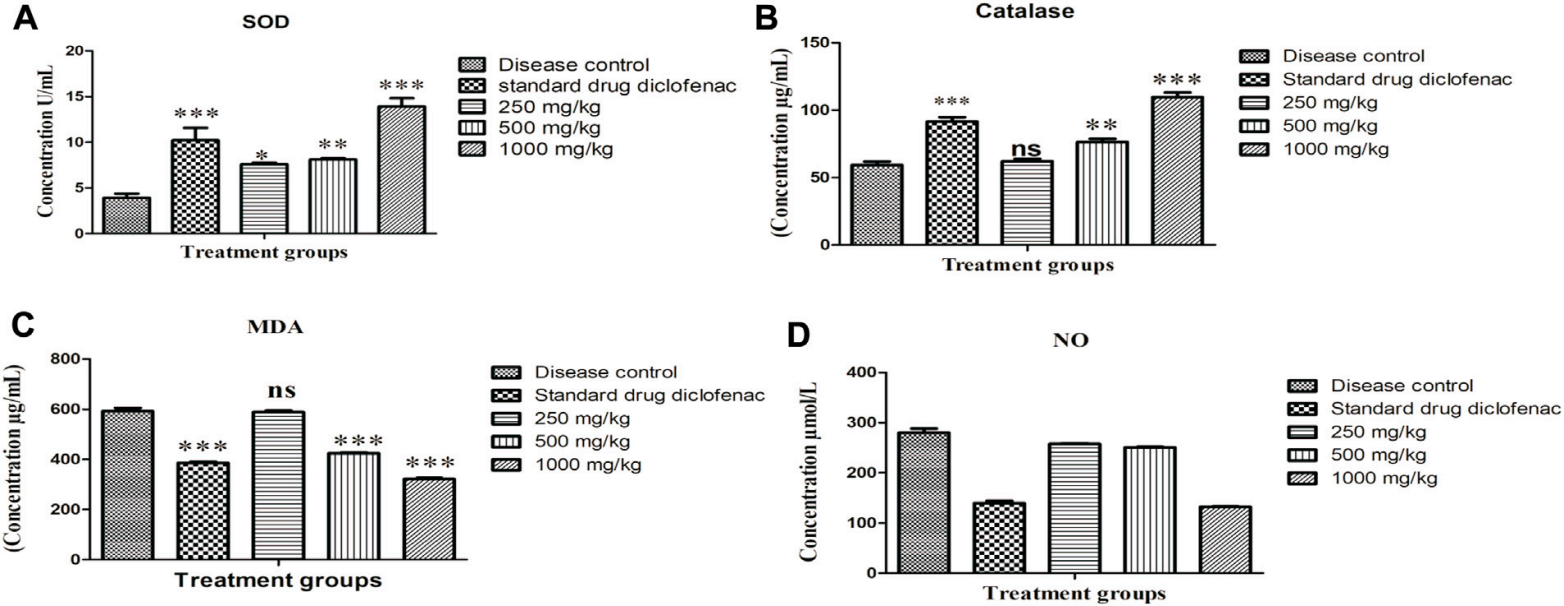
Effect of *Alternanthera bettzickiana* ethanolic extract on oxidative stress biomarkers **(A)** SOD, superoxide dismutase, **(B)** catalase, **(C)** MDA, malondialdehyde, and **(D)** NO, nitric oxide in formaldehyde induced arthritis. Results are presented as mean ± SEM (*n* = 5) and analyzed with one-way ANOVA followed by a Bonferroni posttest. ***p < 0.001; **p < 0.01; * *p* < 0.05; ns, nonsignificant as compared to disease control. ABEE, *Alternanthera bettzickiana* ethanolic extract.

The outcomes of oxidative stress exhibited that SOD (3.93 ± 0.447 U/mL) and CAT concentrations in serum (59.31 ± 2.47 U/L) were significantly reduced (*p* < 0.001) in disease control animals. However diclofenac sodium (10 mg/kg), 500 and 1,000 mg/kg concentration of plant extract increased (*p* < 0.001) the serum concentrations of SOD and CAT in adjuvant-injected groups, as shown in Figure 3A,B respectively. MDA (592.53 ± 12.26 ng/ml) and NO concentrations in serum (279.81 ± 8.518 μmol/L) were also remarkably elevated (*p* < 0.001) in disease control animals. MDA and NO concentrations were significantly reduced by diclofenac sodium and plant extract in adjuvant-injected groups as shown in Figure 3C,D respectively.

## 4 Discussion

Plants have been utilized for the treatment of various disorders for a very long time (Ridtitid et al., 2008). Therapy with medicinal plants is acquiring popularity because WHO supports their proper ethno-medicinal use and calls for the safety assessment of herbal therapy (Daswani et al., 2006; Ogbonnia et al., 2010; Vaghasiya et al., 2011). Who highlights the validation of safe usage of herbal medicines by conducting different toxicity studies? No mortality was found, although some alterations in behavior, such as a decrease in somatomotor activity, elevated respiratory rate, tremor, and itching were noted in acute toxicity group (Table 1). It was noted that water and food intake were normal during the study period, and body weight changes were found to be nonsignificant, which indicates normal protein, lipid, and carbohydrate metabolism inside the body (Zhang et al., 2022). These nutrients are important for performing various physiological functions in human body (Stevens and Mylecraine, 1994; Saleem et al., 2017).

The liver, lungs, kidney, spleen, and heart are major human organs and are important targets sites of any metabolic toxin agent. The metabolic reactions initiated by toxic substances mostly target the heart, kidney, and liver (Vaghasiya et al., 2011). When rats were slaughtered after 14 days, no lesions were observed on macroscopic assessment of liver, heart, and kidney in contrast to vehicle control group. Statistically, no significant alterations were observed in organ-to-body weight index in rats of acute toxicity group in comparison to control group. Substances are classified into five groups on the basis of LD50 values following globally harmonized classification system (Saleem et al., 2017). ABEE can be placed in group 5 because it falls in a lower-toxicity category (LD50 > 2000 mg/kg).

Animal health is assessed by various variables, such as serum biomarker assessment. Liver damage induced by any toxic substance could cause increased total proteins and increased AST and ALT levels (Ozer et al., 2008; Ramaiah, 2011). Statistically nonsignificant increases in globulin, total proteins, and ALT levels were noticed in acute toxicity study. Hepatocellular injury enhances membrane permeability and causes a discharge of aminotransferases in blood (Ali et al., 2008; Ogunlana et al., 2013). ALP is a marker of biliary tract blockage (Manjunatha et al., 2005). In this study, ALP value was significantly reduced (Table 3), which shows hepatoprotective activity of plant (Saleem et al., 2016). Hyperlipidemias are due to other factors like therapies, diet, and disorders, such as tumors, diabetes, hypothyroidism, or nephrosis (Hou et al., 2022). Decreased values of triglycerides, cholesterol, high density lipoprotein, low density lipoprotein, and very low density lipoprotein (Table 3) were observed in acute toxicity group (Saleem et al., 2017; Zhang et al., 2022).

Histopathological assessment is a benchmark for assessing pathological signs exhibited in simultaneous study in which the architecture of organs of treated rats was normal, toxicological signs were not present, and matches with vehicle rats architecture, showing harmless findings of ABEE with LD50 value more than 2000 mg/kg. Toxic substances are metabolically changed into harmless substances in the liver, which is the principal area for metabolism (Harizal et al., 2010; Li et al., 2022). At initial phase signs in hepatocytes are not understandable because of suitability of liver to revive the injurious tissues (Salawu et al., 2009). Histopathological assessment of vital organs did not show any inflammation and degeneration, and only slight congestion was observed in kidney tissues of acute toxicity group. The absence of lesions in liver, kidney, and heart indicate the safety level of plants in experimental animals. The results exhibit that overall no noxious signs were observed during the study period and LD50 was greater than 2000 mg/kg (Saleem et al., 2017).

Formaldehyde-induced arthritis is an appropriate method of assessing the anti-proliferative potential and evaluate anti-arthritic agents. Formaldehyde induces arthritis by breaking down proteins at the site of injection, which produces an immune response against the degraded substances (Nair et al., 2012). Arthritic action of formaldehyde consists of two phases. In the initial phase, substance P is released, while in the late phase bradykinin, histamine, serotonin, and prostaglandin are released, which results in marked permeability and vasodilation (Bischoff, 2008). These mediators are responsible for hyperalgesia by stimulating nerve terminals and pain receptors. Hence, hypersensitivity is evoked at the injection site (Desai Nilesh et al., 2012). It has been revealed that CNS acting drugs hinder both phases uniformly, but peripherally acting drugs hamper the late phase (Uttra and Hasan, 2017).

In the present study, the inhibition of paw edema is due to the potential of *A. bettzickiana* to prevent protein denaturation. It may be due to a decrease in the release of inflammatory mediators. The impediment of both phases of inflammation shows that *A. bettzickiana* acts on CNS. Formaldehyde induces an arthritic model that resembles human arthritis, thus allowing for the evaluation of the anti-arthritic activity of test substance. Formaldehyde injection develops localized inflammation.

TNF-α and IL-6 promote joint damage, bone erosion, and cell death in the inflamed joints (Zheng et al., 2014). The increased formation of TNF-α leads to increase in the expression of IL-6 and IL-1β, which in turn produce the degrading enzymes responsible for osteoclast differentiation, thus stimulating the development of arthritic erosion and vasodilation in the edematous area (Cheng, 2015; Sano, 2011). IL-6 causes bone resorptions and autoantibody production (Alvarez, 2009). Any substance that blocks the production of IL-6 and TNF-α causes a significant revolution in the management of rheumatoid arthritis (Nair et al., 2012).

Reactive oxygen species (ROS) are formed as a consequence of metabolism and different environmental factors, such as cigarette smoke or air pollutants. ROS are produced at low concentrations in all tissues. In general, different antioxidant systems regulate this formation. High concentration or insufficient removal of ROS leads to oxidative stress (Gambhir et al., 1997). ROS are active and can harm cell structures, such as proteins, nucleic acids, lipids, and carbohydrates. They can change their functions. The disturbance in balance between oxidants and antioxidants is called oxidative stress. The control of redox state is important for cell proliferation and cell viability. The antioxidant system is of two types. This includes enzymatic and nonenzymatic antioxidants that are effective in neutralizing damaging effects of ROS (Birben, 2012), although in disease conditions, the antioxidant system can be overcome. Oxidative stress comes up with many disease conditions, such as cancer atherosclerosis, diabetes, asthma, and hypertension. The main enzymatic antioxidants are GSH-px, catalase, and SOD. SOD plays crucial role in free radical defense (Kumar et al., 2016). Glutathione peroxidase and catalase inhibit the accumulation of H_2_O_2_ by changing it into water and oxygen.

Decomposition of lipids produces many products including malondialdehyde (Halliwell, 1991; Baskol et al., 2005). Lipid peroxidation is a well-accepted mechanism of cell damage and it indicates oxidative stress in tissues and is an indicator of lipid peroxidation. Increased levels of MDA are reported in the plasma of rheumatoid arthritis patients (Karatas et al., 2003; Taysi et al., 2002). Nitric oxide is an endogenously formed molecule that possesses important functions in cell signaling and participates in different physiological processes. Radical species that cause oxidative activity in an inflamed joint include reactive oxygen and nitrogen species. These mediate cartilage damage (Ali et al., 2008). NO performs different functions in inflamed areas, such as apoptosis, signal transduction, and mitochondrial function (Phillips et al., 2010). Elevated concentrations of NO in synovial fluid and serum have been reported in rheumatoid arthritis (Ersoy ET., 2010). The current study explains the promising effect of ABEE in formaldehyde-induced arthritis. The results of current study showed that ABEE at dose of 1,000 mg/kg deceased edematous reactions suggesting that constituents of *A. bettzickiana* target biphasic reactions (Table 6).

The outcomes of a previous study showed that ABEE decreased oxidative stress and mRNA expression of pro-inflammatory biomarkers (NF-kB, IL-6, TNF-α, IL-1β, and COX-2), although it increased mRNA expression of immunoregulatory cytokines (IL-4, I-kB, and IL-10). These mechanisms could be accountable for the anti-inflammatory and the anti-arthritic potential of plants. The anti-inflammatory potential of ABEE may be due to the existence of many phytochemicals, such as n-hexadecanoic acid, linoleic acid, phytol, vitamin E, Ar-turmerone, squalene, and farnesol, which possess anti-inflammatory activity (Pamila and Karpagam, 2017b; Manan et al., 2020).

## 5 Conclusion

This study concluded that *Alternanthera bettzickiana* ethanolic extract has low toxicity, as even a 2 g/kg dose did not showed any mortality or morbidity, and no difference was observed in biochemical and hematological parameters in comparison to the control group. Outcomes also proposed that the oral administration of ABEE in arthritic animals remarkably decreased paw thickness and inhibited abnormal changes in hematological and biochemical variables. *Alternanthera bettzickiana* may be used as an optimal therapy for the treatment of rheumatoid arthritis.

## Data Availability

The raw data supporting the conclusion of this article will be made available by the authors, without undue reservation.
